# Total serum bilirubin levels as mediators of anti-atherosclerosis mechanisms with consideration of smoking status

**DOI:** 10.18332/tid/195378

**Published:** 2024-12-02

**Authors:** Shigemasa Tani, Kazuhiro Imatake, Yasuyuki Suzuki, Tsukasa Yagi, Atsuhiko Takahashi

**Affiliations:** 1 Department of Health Planning Center, Nihon University Hospital, Tokyo, Japan; 2 Department of Cardiology, Nihon University Hospital, Tokyo, Japan; 3 Department Medicine, Division of Cardiology, The Nippon Dental University School of Life Dentistry, Tokyo, Japan

**Keywords:** atherosclerosis, cardiovascular disease, bilirubin, smoking, inflammation

## Abstract

**INTRODUCTION:**

Cigarette smoking is a significant risk factor for atherosclerotic cardiovascular diseases (ASCVDs). Mildly elevated total serum bilirubin (TSB) levels can exert anti-atherosclerotic effects and may regulate inflammation. We explore the relationship between TSB levels, smoking, and inflammation.

**METHODS:**

This cross-sectional study evaluated health screening participants with no history of ASCVD between 1 April 2019 and 31 March 2020. TSB was compared between non-smokers and smokers using the Kruskal-Wallis test, and the influencing factors of TSB levels were identified using multivariable logistic regression with TSB levels above the 75th percentile set as the dependent variable. Mediation analysis was performed to determine whether TSB levels mediated the association between smoking and inflammation.

**RESULTS:**

A total of 8337 participants (mean age: 46.6 ± 13.0 years; 58.9% men) were included. TSB levels were significantly lower in smokers (n=1353) than in non-smokers (n=6984) [median (IQR): 0.72 (0.56–0.92) vs 0.76 (0.60–0.97) mg/dL, p<0.0001]. Multivariable logistic regression analysis demonstrated that smoking was an independent determinant of lower TSB levels (adjusted odds ratio, AOR=0.81; 95% CI: 0.70–0.93, p=0.003). Leukocyte counts and C-reactive protein levels significantly decreased as TSB levels increased (p<0.0001). Moreover, the relationship between the duration of smoking cessation and TSB levels showed a positive correlation. Mediation analysis indicated that cigarette smoking had significant indirect effects on higher leukocyte counts and higher CRP levels (coefficient=0.014; 95% CI: 0.008–0.021; and coefficient=0.002; 95% CI: 0.001–0.003, respectively].

**CONCLUSIONS:**

Lower TSB levels related to a smoking habit may be associated with higher inflammation, thereby increasing the ASCVD risk. TSB may regulate inflammation and exert antioxidant effects. Furthermore, smoking cessation may lead to higher TSB levels and lower inflammation.

## INTRODUCTION

Smoke contains many oxidizing and inflammatory substances, and thus, smoke inhalation causes various health problems^1^. Atherosclerotic cardiovascular disease (ASCVD) is the second leading cause of death, next only to malignant neoplasms, in Japan. Further, ischemic heart disease and cerebrovascular disease collectively account for 21.6% of all deaths worldwide^2^. Many epidemiological studies have shown the association between smoking and the incidence of ASCVD^3^.

Oxidation and inflammation are closely related and play critical roles in the development of smoking-induced ASCVD^4^. The endogenous antioxidant total serum bilirubin (TSB) levels are decreased in smokers, as tobacco’s many oxidative and inflammatory substances enter the systemic circulation from the alveoli^5,6^. A meta-analysis by Lan et al.^7^ showed that moderately elevated serum bilirubin levels inhibited ASCVD development. As a pathophysiological state in which the cardiovascular protective effect of bilirubin is significant, Gilbert syndrome, a form of constitutional jaundice with normal liver function and high serum bilirubin levels, is associated with a low incidence rate of myocardial infarction^8^.

Bilirubin is the final metabolite of heme catabolism after hemoglobin reaches the end of its lifespan *in vivo*. In this process, heme is catabolized to biliverdin by haemoxygenase-1 (HO-1), and biliverdin is further catabolized by biliverdin reductase to produce the final metabolite, bilirubin. Thus, TSB levels, which also reflect HO-1 activity, indicate antioxidant activity *in vivo*^9,10^. Bilirubin inhibits the development of atherosclerosis by exerting endogenous antioxidant activity. It regulates lipid oxidation, platelet activation, vascular endothelial function, and inflammatory responses as a signaling molecule, and its role *in vivo* has attracted attention^11,12^.

Cigarette smoking is an indicator of a less health-conscious lifestyle and is thus associated with other unhealthy behaviors. Unhealthy lifestyle habits may increase oxidative stress, which may, in turn, reduce endogenous TSB levels^13^. Conversely, healthy lifestyle habits may reduce oxidative stress and correlate with higher serum bilirubin levels^14^. However, few studies have examined the association among lifestyle (including cigarette smoking), TSB levels, and inflammatory markers in large populations^14,15^.

This cross-sectional study investigates the relationship among TSB, cigarette smoking habits, and inflammatory markers in Japanese people with no history of ASCVD, and to assess the role of TSB as a mediator of the inflammatory response induced by smoking.

We hypothesized that smoking was associated with lower endogenous antioxidant TSB levels, which activated the inflammatory response, and that TSB may also regulate inflammation as a signaling molecule.

## METHODS

### Study design and population

**Figure 1:**
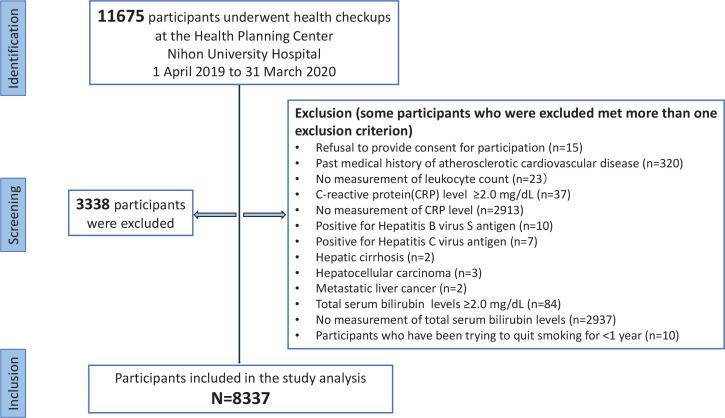
Flow diagram showing inclusion and exclusion criteria for selecting participants in a cross-sectional study conducted at the Health Care Center, Nihon University Hospital, April 2019 – March 2020

A total of 11675 Japanese individuals aged >18 years who underwent annual health checkups between April 2019 and March 2020 at the Health Planning Center of Nihon University Hospital were initially evaluated. Among them, those who met the following criteria were excluded: unwillingness to participate; history of ASCVD; C-reactive protein (CRP) levels ≥2.0 mg/dL; presence of hepatitis B virus surface antigen, hepatitis C antibody, hepatic cirrhosis, hepatocellular carcinoma, and metastatic liver cancer; and lack of measurement of leukocyte, CRP, or TSB levels. Given the potential impact of smoking on TSB, participants who had quit smoking <1 year were accordingly excluded. Further, participants with TSB levels ≥2.0 mg/dL were excluded to avoid the inclusion of individuals with constitutional jaundice as much as possible (i.e. the cutoff was a diagnostic criterion for Gilbert syndrome in the absence of liver dysfunction)^16^. As there were participants in whom TSB levels were higher in fasting blood samples than in random blood samples, we set the cutoff value of TSB levels to be higher than the reference value^17^. Figure 1 shows a flowchart of the patient selection process.

This study was approved by the appropriate institutional review board and complied with the principles of the Declaration of Helsinki. This study was registered as a clinical trial at the University Hospital Medical Information Network Center. The requirement for written informed consent was waived owing to the retrospective cross-sectional nature of this study and the use of an opt-out recruitment procedure.

### Questionnaire for determining healthy behaviors

Participants who underwent health checkups were given a lifestyle questionnaire and asked to respond appropriately. The questions were similar to those in the self-reported questionnaire described in previous studies^18^. Trained interviewers conducted health behavior surveys at our institute via face-to-face interviews with the participants. The surveys comprised comprehensive questions to assess the participants’ demographic and socioeconomic characteristics, including age, occupation, marital status, lifestyle behaviors, current medications, medical history, and family medical history. The questionnaire was a modified version of the Questionnaire on Specific Health Examination, which was used for specific health guidance during health checkups under the Japanese Ministry of Health, Labor and Welfare^19^. The aforementioned questionnaire is an excerpt of questions relevant to this study from our institution’s questionnaire.

The following questions were incorporated into the questionnaire. For smoking habits (current smoking, ex-smoking, or never smoking to date), the participants were asked: ‘Do you smoke habitually?’ with response options ‘No/Yes/I quit smoking/I quit smoking (_) years ago’. Current and ex-smokers were asked about the number of cigarettes smoked per day and in the past, respectively. The participants were asked about their exercise habits with the question: ‘Do you engage in exercise that makes you sweat slightly for 30 minutes a day, at least twice a week for 1 year?’. They were considered to have aerobic exercise habits when light- or moderate-impact exercise (e.g. walking, jogging, aerobics, cycling, swimming) was sustained for sufficiently long periods to require hemoglobin for aerobic metabolism. The presence or absence of intensive daily physical activity was assessed with the question: ‘Do you walk or engage in similar physical activity for 1 h or more per day in your daily life?’ with response ‘Yes or No’. Drinking habits were assessed with the question: ‘Please indicate the frequency at which you drink alcohol’ with response ‘Every day/sometimes/I used to drink previously, but have stopped drinking/I stopped drinking (_) years ago/I rarely drink/I cannot drink’ and ‘How much do you drink per day when you drink?’ with response (ethanol equivalent [g/day]): <20 g; 20 g to <40 g; 40 g to <60 g, ≥60 g. How many days per week do you drink?’. Daily sleeping habits were assessed using the questions: ‘How many hours do you sleep daily, on average?’ and ‘Do you feel adequately rested in the morning after sleeping?’.

### Physical examination and laboratory parameters

Anthropometric variables (height and weight) were measured using standardized techniques and equipment while the participant was standing. Waist circumference was measured at the umbilicus level with a non-stretchable tape measure during the late exhalation phase with the participant standing. Following a 5-minute rest period, blood pressure was measured twice (with a 3-minute interval between measurements) using a standard mercury sphygmomanometer, and the average was used for our analysis. Blood samples were collected early in the morning after the participants had fasted for 8 h. TSB levels were determined using an autoanalyzer (Hitachi Corporation, Tokyo, Japan). The degree of day-to-day precision [coefficient of variation (CV) for TSB ranged from 1.2% (206.2 mg/L) to 10.6% (3.5 mg/L)]^20,21^. Blood leukocyte counts were determined using a Beckman Coulter STKS (Beckman Coulter Inc., Fullerton, CA, USA) analyzer. Serum CRP levels were measured using a nephelometric assay (Behring Diagnostics, Marburg, Germany). Enzymatic assays measured serum total cholesterol, triglyceride, and high-density lipoprotein cholesterol levels. Low-density lipoprotein cholesterol serum levels were measured using the Friedewald formula^22^. Glycated hemoglobin A1c levels were measured using high-performance liquid chromatography^23^.

### Statistical analysis

Data are expressed as mean ± standard deviation (SD) for continuous variables and as frequencies and percentages for discrete variables. The normality of the distribution of continuous variables was assessed using the Kolmogorov-Smirnov and Shapiro-Wilk tests. Variables with a significantly skewed distribution are presented as median and interquartile range (IQR). Comparisons between groups were performed using the chi-squared test for categorical data and the Kruskal–Wallis test for non-parametric data. Trend analysis was used to compare multiple groups. As appropriate, linear trends in participant characteristics according to the TSB level quartiles were analyzed using either the Jonckheere–Terpstra or the Cochran–Armitage trend test. To identify factors influencing TSB levels, we performed univariable and multivariable logistic regression analyses with the TSB levels above the 75th percentile as the dependent variable. We set the dependent variable at the 75th percentile for convenience to emphasize that a higher value of TSB was associated with a reduced risk of ASCVD (i.e. an ASCVD low-risk group) and that even in the ASCVD low-risk group, smoking increased the risk of ASCVD. The independent variables included statistically significant factors (p<0.05) according to the TSB level quartiles in the linear trend test. Significant factors in the univariable logistic regression analysis were then entered into the multivariable logistic regression models. As the TSB levels were not normally distributed, non-parametric correlation coefficients (Spearman’s rank correlation coefficient) were used. All statistical analyses were performed using the Statistical Package for the Social Sciences (SPSS), version 29.0 (IBM Corp., Armonk, NY, USA). A two-sided p<0.05 was considered statistically significant.

### Mediation analysis

**Figure 2:**
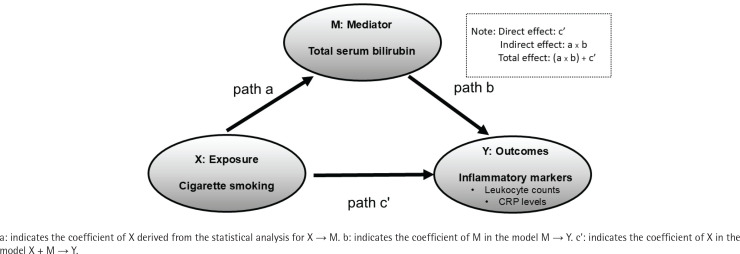
Patient selection flowchart, in a cross-sectional study conducted at the Health Care Center, Nihon University Hospital, April 2019 – March 2020 (N=8337)

The mediation model helps explain the relationship between an exposure variable (cigarette smoking) and an outcome measure (leukocyte counts and CRP levels) through a third (mediator) variable (TSB levels)^24^. Briefly, the mediation model (Figure 2) is a series of regression analyses involving three paths: 1) path a is a regression between the exposure and the mediator; 2) path b is a regression between the mediator and the outcome, adjusting for the exposure; and 3) path c’ is a regression between the exposure and the outcome, adjusting for the mediator. In the mediation model, the products of ab and c’ are mathematically equivalent, and ab is the ‘amount’ of mediation or contribution that a mediator makes to the relationship between an exposure and an outcome. For the mediation analysis, we used Hayes’ PROCESS macro for mediation, moderation, and conditional process analysis in SPSS with 5000 iterations to estimate the amount of mediation or contribution (ab) by each mediator. We present the bias-corrected ‘ab’ estimates with 95% confidence intervals (CI)^25,26^.

## RESULTS

### Participant characteristics

After excluding 3338 participants, 8337 participants were included in the analysis. Men and women accounted for 58.9% (n=4914) and 41.1% (n=3423) of the cohort, respectively. The mean participant age was 46.6 ± 13.0 years [men: 48.2 ± 12.7 years (range: 18–89 years), women: 44.2 ± 13.1 years (range: 19–92 years)]. Current smokers comprised 16.2% (n=1353) of the cohort; ex-smokers, 19.5% (n=1628); and never smokers, 64.2% (n=5357).

### Comparison of total serum bilirubin levels between current smokers and non-smokers

**Figure 3:**
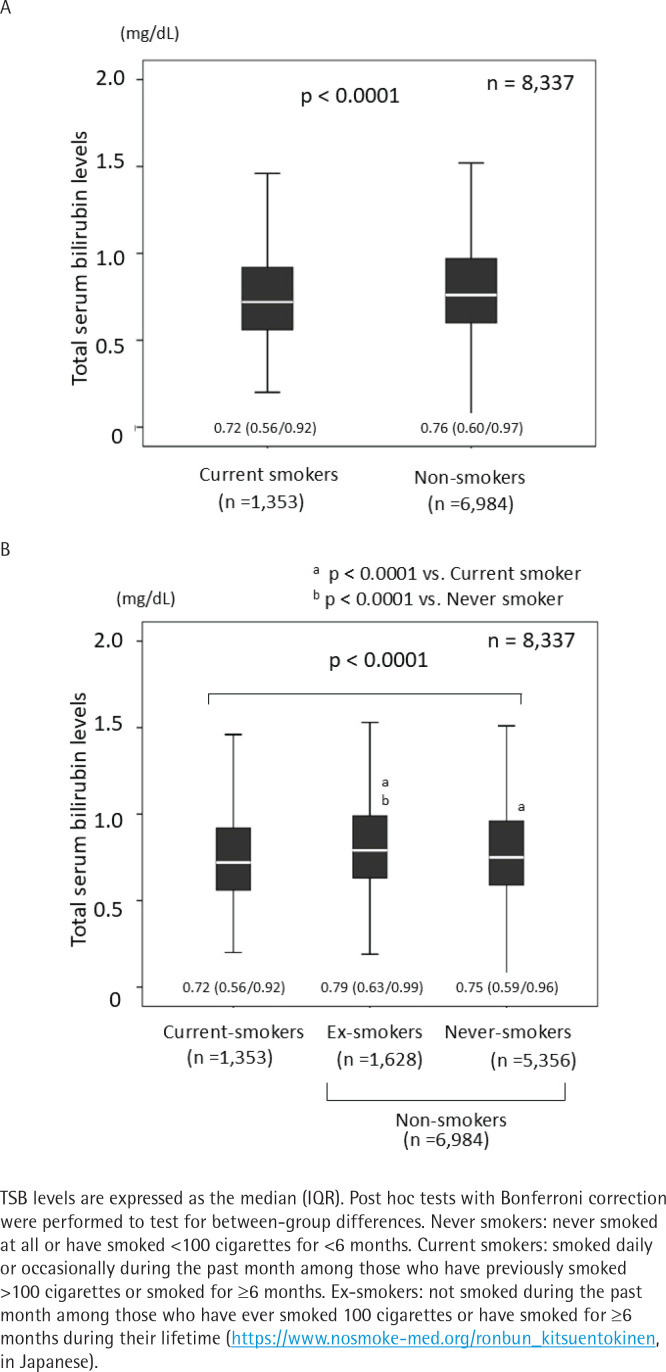
Comparison of total serum bilirubin levels: A) between current smokers and non-smokers; B) between current smokers, ex-smokers, and never smokers, among survey participants in a crosssectional study conducted at the Health Care Center, Nihon University Hospital, April 2019 – March 2020 (N=8337)

TSB levels in current smokers were significantly lower than those in non-smokers (p<0.0001) (Figure 3A). TSB levels also differed significantly among current, ex-smokers, and never smokers, with significantly lower TSB levels in current smokers than in ex-smokers and never smokers (p<0.0001). Interestingly, TSB levels were significantly higher in ex-smokers than in never smokers (Figure 3B).

### Comparison of participant characteristics, inflammatory markers, and lifestyle according to total serum bilirubin levels

Participants engaging in habitual aerobic exercise and intensive daily physical activity showed significantly higher TSB levels (p<0.0001 and p=0.007, respectively). Higher TSB levels were also significantly associated with feeling adequately rested after sleeping (p=0.007) and with longer sleeping time (p=0.010). No significant associations were observed between TSB levels and other variables (Table 1).

**Table 1:** Comparison of participant characteristics, laboratory data, and lifestyle behaviors according to total serum bilirubin levels, among survey participants in a cross-sectional study conducted at the Health Care Center, Nihon University Hospital, April 2019 – March 2020 (N=8337)

*Characteristics*	*All cases* *(N=8337)*	*TSB quartiles*				*p for trend
		*Q 1* *(N=2008)*	*Q 2* *(N=2098)*	*Q 3* *(N=2104)*	*Q 4* *(N=2127)*	
**TSB level** (mg/dL), median (IQR)	0.75 (0.07–1.98)	0.50 (0.07–0.58)	0.66 (0.59–0.74)	0.83 (0.75–0.95)	1.14 (0.96–1.98)	
**Age** (years), mean ± SD	46.6 ± 13.0	46.6 ± 12.9	46.5 ± 13.0	46.7 ± 13.0	46.5 ± 13.1	0.966
**Male gender**, n (%)	4914 (58.9)	1219 (60.7)	1233 (58.8)	1222 (58.1)	1240 (58.3)	0.106
**ASCVD risk,** mean ± SD						
Waist circumference (cm)	81.7 ± 10.4	82.0 ± 10.4	81.8 ± 10.5	81.4 ± 10.4	81.8 ± 10.3	0.307
Body mass index (kg/m^2^)	23.3 ± 3.7	23.4 ± 3.8	23.3 ± 3.8	23.2 ± 3.7	23.3 ± 3.7	0.972
Visceral obesity, n (%)	2651 (31.8)	673 (33.5)	661 (31.5)	631(30.0)	683 (32.1)	0.114
TC (mg/dL)	201 ± 34	201 ± 34	201 ± 34	201 ± 33	202 ± 36	0.529
LDL-C (mg/dL)	117 ± 30	117 ± 30	117 ± 30	118 ± 30	117 ± 30	0.664
HDL-C (mg/dL)	62 ± 16	62 ± 16	61 ± 16	62 ± 16	62 ± 16	0.257
TG (mg/dL), median (IQR)	81 (57–120)	82 (57–121)	81 (58–122)	80 (57–118)	82 (57–120)	0.644
non-HDL-C (mg/dL)	139 ± 34	139 ± 34	139 ± 34	139 ± 33	139 ± 35	0.968
Systolic blood pressure (mmHg)	118 ± 15	118 ± 14	118 ± 15	118 ± 15	118 ±15	0.653
Diastolic blood pressure (mmHg)	75 ± 12	76 ± 12	75 ± 11	75 ± 12	75 ± 12	0.356
Pulse rate (bpm)	72 ± 11	72 ± 11	72 ± 11	72 ± 11	72 ± 11	0.89
FBG (mg/dL)	99 ± 17	99 ± 17	99 ± 18	99 ± 17	99 ±18	0.783
HbA1c (%)	5.7 ± 0.6	5.7 ± 0.6	5.7 ± 0.6	5.7 ± 0.6	5.8 ± 0.6	0.774
Diabetes mellitus, n (%)	650 (7.8)	159 (7.9)	183 (8.7)	149 (7.1)	157 (7.4)	0.231
Uric acid (mg/dL)	5.6 ± 1.4	5.7 ± 1.4	5.7 ± 1.4	5.6 ± 1.4	5.6 ± 1.4	0.507
**Inflammatory markers,** median (IQR)						
Leukocyte count (cells/μL)	4700 (4000–5600)	4900 (4100–5900)	4700 (4000–5600)	4700 (4000–5600)	4600 (3900–5500)	<0.0001
CRP (mg/dL)	0.04 (0.02–0.09)	0.05 (0.02–0.12)	0.04 (0.02–0.10)	0.03 (0.02–0.08)	0.03 (0.02–0.07)	<0.0001
**Drug treatment,** n (%)						
Anti-hypertensive drug	1179 (14.1)	251 (12.5)	320 (15.3)	313 (14.9)	295 (13.9)	0.299
Anti-diabetic drug	330 (4.0)	81 (4.0)	98 (4.7)	80 (3.8)	71 (3.3)	0.116
Lipid-modifying drug	716 (8.6)	175 (8.7)	200 (9.5)	170 (8.1)	171 (8.0)	0.119
Antihyperuricemic drug	354 (4.2)	80 (4.0)	101 (4.8)	83 (3.9)	90 (4.2)	0.934
**Cigarette smoking status,** n (%)						
Cigarette smoker	1353 (16.2)	378 (18.8)	343 (16.3)	333 (15.8)	299 (14.1)	<0.0001
Former smoker	1628 (19.5)	352 (17.5)	410 (19.5)	440 (20.9)	426 (20.0)	0.025
Never smoker	5356 (64.2)	1305 (65.0)	1345 (64.1)	1322 (62.8)	1384 (65.1)	0.844
**Lifestyle behaviors,** n (%)						
Aerobic exercise habit	2140 (25.7)	450 (22.4)	511 (24.4)	581 (27.6)	598 (28.1)	<0.0001
Intensive daily physical activity	3655 (43.8)	845 (42.1)	882 (42.5)	946 (45.0)	972 (45.7)	0.046
Sleep duration <4 h/day	390 (4.7)	116 (5.8)	98 (4.7)	88 (4.2)	88 (4.1)	0.046
Feeling refreshed after sleep	5147 (61.7)	1180 (58.8)	1295 (61.7)	1309 (62.2)	1363 (64.1)	0.005
Intake >400 g ethanol/week	124 (1.5)	27 (1.3)	26 (1.2)	33 (1.6)	38 (1.8)	0.466

* Comparisons among multiple groups are performed using trend analysis, and linear trends in participant characteristics are analyzed according to the TSB level quartiles using either the Jonckheere–Terpstra test or Cochran–Armitage trend test. TSB: total serum bilirubin. TC: total cholesterol. LDL: low-density lipoprotein. HDL: high-density lipoprotein. TG: triglyceride. BP: blood pressure. FBG: fasting blood glucose. IQR: interquartile range. Aerobic exercise habit: aerobic exercise >30 minutes at least twice weekly. Intensive daily physical activity: walking or engaging in similar physical activity for ≥1 h daily. Average weekly alcohol intake: number of alcoholic drinks/week × amount of alcohol consumed/drink (ethanol equivalent, g/week); in Japan, we roughly convert ethanol intake into *go*, an index of the amount of Japanese sake consumed, where 180 mL of sake (1 *go*) equals approximately 20 g of ethanol; alcoholic beverage equivalent of 1 *go* (20 g ethanol) is 500 mL of beer, 180 mL of wine, or 60 mL of whiskey.

### Influencing factors of total serum bilirubin levels

**Figure 4:**
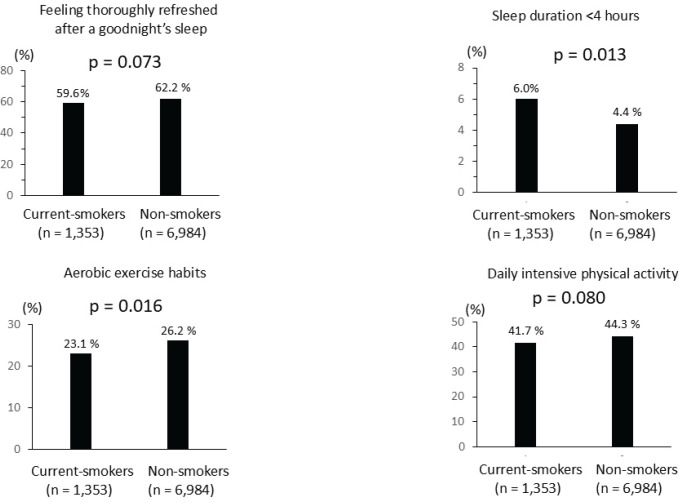
Cigarette smoking habits and lifestyle, among survey participants in a cross-sectional study conducted at the Health Care Center, Nihon University Hospital, April 2019 – March 2020 (N=8337)

Univariate logistic regression analysis showed that smoking and sleeping less than 4 h/day were negative determinants of lower TSB levels. Conversely, aerobic exercise habits, feeling fully rested after sleeping, and intensive daily physical activity were positive determinants of higher TSB levels. These factors were included in the multivariable logistic regression analysis. Current smoking was a significant negative determinant of lower TSB level (AOR=0.81; 95% CI: 0.71–0.93, p=0.003). Meanwhile, aerobic exercise (AOR=1.14; 95% CI: 1.02–1.29, p=0.025) and feeling adequately rested after sleeping (AOR=1.12; 95% CI: 1.01–1.24, p=0.04) were independent factors influencing high TSB levels. However, sleep duration of less than 4 h/day and intensive daily physical activity were not independent determinants of TSB (Table 2). We further examined smoking and lifestyle behaviors. As shown in Figure 4, participants with smoking habits were less likely to have healthy lifestyles compared with those without smoking habits. These results indicated that cigarette smoking was associated with unhealthy lifestyle habits.

**Table 2:** Univariable and multivariable logistic regression analysis to identify factors influencing total serum bilirubin levels, among survey participants of a cross-sectional study conducted at the Health Care Center, Nihon University Hospital, April 2019 – March 2020 (N=8337)

*Variables*	*Univariable*				*Multivariable*			
	*OR*	*95% CI*		*p*	*AOR*	*95% CI*		*p*
		*Lower*	*Upper*			*Lower*	*Upper*	
Current cigarette smoker	0.80	0.70	0.92	0.002	0.81	0.71	0.93	0.003
Aerobic exercise habit	1.19	1.06	1.33	0.002	1.17	1.04	1.30	0.004
Feeling refreshed after sleep	1.15	1.03	1.27	0.009	1.13	1.02	1.25	0.023
Sleep duration <4 h/day	0.84	0.66	1.17	0.171				
Intensive daily physical activity	1.11	1.00	1.22	0.430				

AOR: adjusted odds ratio. Univariable and multivariable logistic regression analyses were performed with the TSB levels above the 75th percentile in the study participants as the dependent variable. The independent variables include statistically significant factors (p<0.05) according to the TSB level quartiles in the linear trend test. Factors with p<0.05 in univariable logistic regression analysis were entered into multivariable logistic regression models.

### Comparison of inflammatory markers between current smokers and non-smokers

The levels of inflammatory markers in smokers were significantly higher than those in non-smokers [median leukocyte counts: 5300 (4500–6300) cells/μL vs 4600 (3900–5500) cells/μL, p<0.0001; CRP levels: 0.05 (0.02–0.23) mg/dL vs 0.04 (0.02–0.08) mg/dL, p<0.0001).

### Relationship between duration of smoking cessation and total serum bilirubin levels

**Figure 5:**
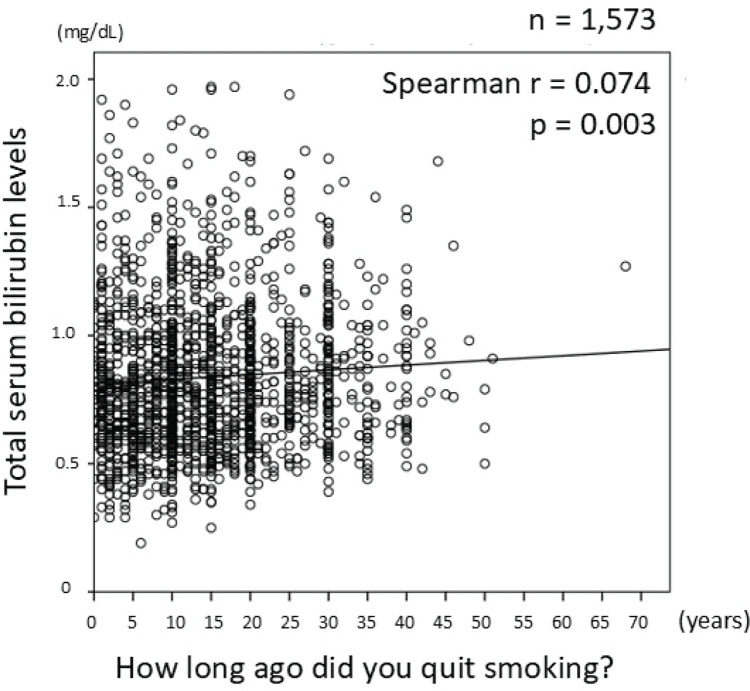
Relationship between duration after smoking cessation and total serum bilirubin levels, among survey participants in a cross-sectional study conducted at the Health Care Center, Nihon University Hospital, April 2019 – March 2020 (N=8337)

Analysis of the relationship between the duration of smoking cessation and TSB levels showed a positive correlation (Spearman r=0.074, p=0.003); TSB levels increased after participants quit smoking (Figure 5). Therefore, multiple regression analyses were conducted in participants who had quit smoking, with TSB as the dependent variable and the duration of quitting smoking, aerobic exercise, and feeling rested after sleeping as the independent variables. The results showed that a longer duration of quitting smoking was the only independent determinant of higher TSB levels (β=0.062, p=0.0014).

### Role of TSB as a mediator of smoking-induced inflammatory response

Table 3 presents the estimates of the mediation or contribution of TSB levels to the association between cigarette smoking and inflammatory markers. The total effects of cigarette smoking on higher leukocyte counts and higher CRP levels were significant (coefficient=0.745; 95% CI: 0.670–0.820, and coefficient=0.012; 95% CI: 0.002–0.022, respectively). The indirect effects of cigarette smoking on higher leukocyte counts and higher CRP levels were also significant (coefficient=0.014; 95% CI: 0.008–0.021, and coefficient=0.002; 95% CI: 0.001–0.003, respectively). In contrast, the direct effect was significant for higher leukocyte counts but not for higher CRP levels (coefficient=0.731; 95% CI: 0.656–0.806, and coefficient=0.0096; 95% CI: -0.0003–0.019, respectively). Further, lower CRP levels with no cigarette smoking may be mainly influenced by higher TSB levels as a mediator. In contrast, lower leukocyte counts with no cigarette smoking may be associated with a direct effect of not smoking and higher TSB levels as a mediator. Particularly, smoking itself was associated with higher leukocyte counts and CRP levels. However, higher leukocyte counts were relevant to both the direct effect of smoking and the indirect effect via TSB. In contrast, higher CRP levels were associated only with the indirect effect via TSB.

**Table 3:** Mediation effects of total serum bilirubin levels on the association between cigarette smoking and inflammatory markers, among survey participants in a cross-sectional study conducted at the Health Care Center, Nihon University Hospital, April 2019 – March 2020 (N=8337)

*Inflammatory marker (Y)*	*X → M* *(a)*	*M → Y* *(b)*	*Direct effect* *(c’)*	*Indirect effect* *(ab)*		*Total effect* *(ab + c’)*
	*Coefficient* *(95% CI)*	*Coefficient* *(95% CI)*	*Coefficient* *(95% CI)*	*Coefficient* *(95% CI)*	*Proportion %*	*Coefficient* *(95% CI)*
Leukocyte counts	-0.04 (-0.06 – -0.03)*	-0.32 (-0.42 – -0.23)*	0.73 (0.66–0.81)*	0.014 (0.008–0.02)*	1.88	0.75 (0.67–0.82)*
CRP levels	-0.04 (-0.06 – -0.03)*	-0.05 (-0.06 – -0.04)*	0.01 (-0.0003–0.02)	0.002 (0.001–0.003)*	16.7	0.012 (0.002–0.022)*

The letters X, M, and Y denote the independent variable (X for current cigarette smoking), mediator (M for TSB), and dependent variable (Y for leukocyte count and CRP levels). The letters a, b, and c’ denote coefficients of X, M, and X in their respective models shown in Figure 1. The proportion of indirect effect to total effect [ab/(ab + c’)] was calculated.

* p<0.05 indicates a statistically significant difference.

## DISCUSSION

This study found that smokers had lower TSB levels, higher leukocyte counts, and higher CRP levels than non-smokers. Furthermore, TSB regulated inflammation, but their role as mediators depended on inflammatory markers. In non-smokers, higher TSB levels may only partly mediate lower leukocyte counts but fully mediate lower CRP levels. These results suggested that cigarette smoking may be associated with lower TSB levels and inflammation, leading to an increased risk of ASCVD. Furthermore, by controlling inflammation, higher TSB levels may help reduce the risk of ASCVD.

Mediation analysis may reveal the potency of different inflammatory pathways induced by smoking. Lower TSB levels in smokers are associated with higher WBC counts and CRP levels, which are predictive inflammatory markers of ASCVD development^27^, indicating the significance of a lower TSB level as a risk factor for ASCVD in smokers. Bilirubin is an effective endogenous antioxidant that scavenges reactive oxygen radicals. Experimental studies have reported that bilirubin regulates several factors that inhibit atherosclerosis development^10,28^. Therefore, increased physiological serum bilirubin levels have been hypothesized to possibly reduce atherogenic risk. Consequently, it is unsurprising that TSB was a mediator of smoking and inflammatory markers in the mediation analysis performed in the current study.

Akboga et al.^29^ investigated the relationship between the Gensini score, an indicator of the degree of coronary atherosclerosis, and TSB levels in patients with coronary artery disease. The results showed that the higher the Gensini score, the lower the TSB levels and that TSB levels were negatively correlated with inflammatory markers^30^. Our findings partly support these results. Cigarette smoking promotes the development of atherosclerosis through several mechanisms. These include the promotion of abnormal lipid metabolism, increased insulin resistance, vascular endothelial damage, and activation of blood coagulation^2^. From another perspective, cigarette-smoking-induced decreases in TSB levels may be a risk factor for ASCVD. Cigarette smoke may contain many oxidants, leading to lower antioxidants in the body and, consequently, lower TSB levels^31^. Furthermore, cigarette smoking can suppress the *HO-1* gene and its protein, with the repressor transcription factor Bach1 found in rat fibroblasts and lung cells^32^. Thus, cigarette smoking may also lower bilirubin levels by suppressing the expression of the *HO-1* gene.

Consistent with a previous finding^33^, we showed that unhealthy lifestyle habits were more common in smokers than in non-smokers. The lower TSB levels in smokers than in non-smokers in the current study may be related to the increased oxidative stress caused by both cigarette smoking and the unhealthy lifestyle habits associated with cigarette smoking, either synergistically or in an additive manner. Aerobic exercise^34^ and good sleep quality^14^ are healthy lifestyle habits that lead to higher TSB levels and significantly help reduce oxidative stress. Thus, they have been advocated as preventive measures against ASCVD^35^. Our findings on the association between a healthy lifestyle and higher TSB levels support this recommendation. Quitting smoking can prevent smoking-related health problems.

As shown in Figure 5, TSB levels gradually start to increase after smoking cessation, and the increase in TSB levels is also due to the synergistic effect of improvements in lifestyle habits other than smoking cessation. Further, ex-smokers had higher TSB levels than did never smokers. Trying to quit smoking may also change other unhealthy lifestyle habits. A cross-sectional study of Belgian women showed higher TSB levels in ex-smokers than in never smokers, indicating that good lifestyle habits are encouraged with smoking cessation^8^. Future prospective cohort studies are needed to establish the target bilirubin level to reduce the risk of ASCVD development. In addition, as bilirubin levels vary widely among individuals, people should also be educated and encouraged to engage in healthy lifestyle habits that improve bilirubin levels.

### Limitations

This study had some limitations. First, we did not demonstrate a relationship between cigarette smoking, TSB levels, and imaging findings of the degree of atherosclerosis progression. Second, non-smokers whose TSB levels were reduced by secondhand smoke could not be excluded from the study population. Third, it was impossible to determine the number of cigarettes smoked daily by all participants. This could be examined more thoroughly if the Brinkman Index^36^ could be calculated. Fourth, we cannot justify setting the dependent variable in the multivariate logistic regression analysis at > 75th percentile of TSB for all study participants (low ASCVD risk group). Fifth, in the present study we performed a mediation analysis and showed some causal relationships among smoking, TSB, and inflammatory markers owing to the study’s cross-sectional nature. Sixth, the participants were health checkup recipients and were healthy. Moreover, patients with a history of ASCVD with multiple risks of atherosclerosis were excluded. Therefore, selection bias could not be ruled out, and thus, the results may not apply to the total Japanese population or those from overseas. Finally, mediation analysis involves several methods, and it is difficult to completely exclude bias due to the many confounding factors for paths ‘a’ and ‘b’. Particularly, the mediation analysis used in this study was not adjusted for confounding factors related to smoking, TSB levels, and inflammatory markers. This could explain why our study demonstrates that TSB is a signal compound and mediator of the causal relationship between cigarette smoking and inflammation. A simple statistical analysis method is needed in future studies.

## CONCLUSIONS

Cigarette smoking is associated with lower TSB levels and higher inflammatory marker levels. Unhealthy lifestyle habits corresponding to cigarette smoking habits may also be associated with lower TSB levels. These habits may be associated with lower TSB levels and more severe inflammation. These findings support the hypothesis that smoking habits are related to lower levels of endogenous antioxidant TSB levels, which activate the inflammatory response and lead to higher ASCVD risk. Mediation analysis may help estimate the inflammatory processes associated with cigarette smoking. Further studies are required to clarify the causality between these factors.

## Data Availability

Data supporting the findings of this study are available upon request from the corresponding author.
